# Fc-gamma receptor polymorphisms as predictive and prognostic factors in patients receiving oncolytic adenovirus treatment

**DOI:** 10.1186/1479-5876-11-193

**Published:** 2013-08-21

**Authors:** Mari Hirvinen, Raita Heiskanen, Minna Oksanen, Saila Pesonen, Ilkka Liikanen, Timo Joensuu, Anna Kanerva, Vincenzo Cerullo, Akseli Hemminki

**Affiliations:** 1 Cancer Gene Therapy Group, Department of Pathology and Transplantation laboratory, Haartman Institute, University of Helsinki, Haartmaninkatu 3, Helsinki 00290, Finland; 2 Laboratory of Immunovirotherapy, Division of Biopharmaceutics and Pharmacokinetics, Faculty of Pharmacy, University of Helsinki, Viikinkaari 5 E, Helsinki 00790, Finland; 3 Oncos Therapeutics, Ltd., Helsinki, Finland; 4 Docrates Cancer Center, Helsinki, Finland; 5 Department of Obstetrics and Gynecology, Helsinki University Central Hospital, Helsinki, Finland

**Keywords:** Fc gamma receptor (FcgR), Oncolytic virus, NK cell, GM-CSF, CD40L

## Abstract

**Background:**

Oncolytic viruses have shown potential as cancer therapeutics, but not all patients seem to benefit from therapy. Polymorphisms in Fc gamma receptors (FcgRs) lead to altered binding affinity of IgG between the receptor allotypes and therefore contribute to differences in immune defense mechanisms. Associations have been identified between FcgR polymorphisms and responsiveness to different immunotherapies. Taken together with the increasing understanding that immunological factors might determine the efficacy of oncolytic virotherapy we studied whether FcgR polymorphisms would have prognostic and/or predictive significance in the context of oncolytic adenovirus treatments.

**Methods:**

235 patients with advanced solid tumors were genotyped for two FcgR polymorphisms, FcgRIIa-H131R (rs1801274) and FcgRIIIa-V158F (rs396991), using TaqMan based qPCR. The genotypes were correlated with patient survival and tumor imaging data.

**Results:**

In patients treated with oncolytic adenoviruses, overall survival was significantly shorter if the patient had an FcgRIIIa-VV/ FcgRIIa-HR (VVHR) genotype combination (*P* = 0,032). In contrast, patients with FFHR and FFRR genotypes had significantly longer overall survival (*P* = 0,004 and *P* = 0,006, respectively) if they were treated with GM-CSF-armed adenovirus in comparison to other viruses. Treatment of these patients with unarmed virus correlated with shorter survival (P < 0,0005 and P = 0,016, respectively). Treating FFHH individuals with CD40L-armed virus resulted in longer survival than treatment with other viruses (P = 0,047).

**Conclusions:**

Our data are compatible with the hypothesis that individual differences in effector cell functions, such as NK cell-mediated antibody-dependent cellular cytotoxicity (ADCC) and tumor antigen presentation by APCs caused by polymorphisms in FcgRs could play role in the effectiveness of oncolytic virotherapies. If confirmed in larger populations, FcgR polymorphisms could have potential as prognostic and predictive biomarkers for oncolytic adenovirus therapies to enable better selection of patients for clinical trials. Also, putative associations between genotypes, different viruses and survival implicate potentially important mechanistic issues.

## Background

Oncolytic viruses are modified to selectively kill cancer cells by replicating in them. Replicating viruses have shown promising efficacy in human clinical trials as cancer therapeutics but the treatment is usually not curative, especially in the context of advanced cancer [1]. Immunological factors are currently believed to play a crucial role in determining the efficacy of the treatment or the lack thereof [2,3]. Adenoviruses per se are known to be highly immunogenic but it seems that even stronger stimulation of patient’s own immune system is required to fully overcome the immunosuppressive tumor microenvironment and to ensure a long-lasting effect. In this regard, boosting the immunogenicity of the virus e.g. by inserting immunostimulatory transgenes has shown intriguing results [4,5]. However, patients receiving oncolytic virotherapy seem to respond differently to the treatments, some benefiting more than the others. Individual differences in the efficiency of the patients’ immune system and antigen recognition might play a crucial role in determining the response to viral treatment. However, currently there are no biomarkers available for selecting the right patients for such therapy or vice versa.

Fc gamma receptors (FcgRs) have been recognized as key players in immune defense mechanisms against foreign cells and pathogens. In previous studies, single nucleotide polymorphisms in FcgRIIa and FcgRIIIa genes have not only been associated with susceptibility to various diseases such as autoimmune disorders [6,7], infectious diseases [8] and coagulation defects [9], but also with disease progression and responsiveness to immunological therapies, such as mAb therapies [10,11] and cancer vaccinations [12,13]. For example, Musolino et al. showed association between FcgRIIa and FcgRIIIa polymorphisms and clinical outcome of trastuzumab treated breast cancer patients [11], where individuals with VV and HH genotypes had better survival and response rates compared to patients with other genotypes. Similar results were seen in rituximab treated B-cell lymphoma patients [14,15]. Cetuximab therapy for colorectal cancer was also seen to be more effective for individuals with HH genotype [16,17], although there has been conflicting data published for the allele giving the best outcome [16,18,19].

Polymorphisms in FcgRIIa and FcgRIIIa have also been shown relevant for cancer vaccines. A positive correlation was observed between the outcome of patients treated with idiotype vaccination against B-cell lymphoma and the FcgRIIIa-VV genotype [20]. FcgRIIa-131R and FcgRIIIa-158 V allotypes were associated with significantly better overall in survival of colorectal carcinoma patients receiving either passively administered monoclonal antibodies or antibodies induced by carcinoembryonic antigen (CEA) vaccination [13]. Because oncolytic adenoviruses are known to elicit strong immune reactions in patients [21], and it is increasingly understood that immunological factors determine the efficacy of the treatment or lack thereof [3], we wanted to assess if FcgR polymorphisms play a role.

FcgRs are glycoproteins that are part of the immunoglobulin superfamily expressed on leukocytes. These receptors have a major role in antigen recognition during immune response. They serve as a link between humoral and cell-mediated immune systems, but also between innate and adaptive immune responses [22]. FcgRs can be divided into three classes, FcgRI, FcgRII and FcgRIII, which differ in structure, cell distribution and also in affinity and specificity to different IgG subtypes. Each of these receptor classes can in turn be split into subclasses, indicated with letters (a, b or c). In contrast to FcgRI, which exhibits high affinity to monomeric IgG, FcgRII and FcgRIII are capable of binding effectively only aggregated, multimeric IgG that is bound to an antigen [23]. Binding of antigen-bound IgG leads to activation of FcgRs through phosphorylation of the ITAM/ITIM signaling unit by tyrosine kinases leading to activation of various downstream targets, eventually resulting in changes in effector cell functions. Effector cells bearing FcgRs can destroy target Ag-Ab complexes by multiple mechanisms. FcgRs can regulate effector cell functions including phagocytosis, degranulation, antibody-dependent cellular cytotoxicity (ADCC), cytokine and chemokine expression or antibody production by B-cells [23-25].

At least eleven polymorphic regions can be found in FcgR genes, and copy number variation has also been reported [26]. Among different subclasses, FcgRIIa and FcgRIIIa play the most important role in immune regulation and their polymorphisms have been most often associated with diseases and disease severity in previous studies.

FcgRIIa (CD32a) is one of the three possible receptor subclasses expressed from *FCGRII* genes. It has low affinity to monomeric IgG, but binds effectively to aggregated IgG and Ag-Ab immune complexes. FcgRIIa is expressed on monocytes, macrophages, neutrophils, certain dendritic cells (DCs) and also on platelets, which is unique among all FcgRs [22]. FcgRIIa has a wide cellular distribution, but it is mainly expressed on phagocytic cells such as macrophages and neutrophils, and is commonly involved in the process of phagocytosis and clearance of immune complexes [27]. A single nucleotide polymorphism (SNP) in FcgRIIa, FcgRIIa-H131R (rs1801274), alters the binding affinity of the receptor to IgG between the two possible receptor allotypes. In this polymorphism, a missense mutation (guanine (G) - > adenine (A)) in *FCGRIIA* gene results in amino acid change, histidine (H) to arginine (R), in amino acid position 131 in the receptor’s extracellular ligand-binding domain. The FcgRIIa-R allotype binds weaker to IgG (especially to IgG2) than the ancestral H allotype. This variation leads to functional difference between the two allotypes. Effector cells of homozygous individuals for FcgRIIa-H are more effective in recognizing and clearing IgG2-coated antigens than cells of FcgRIIa-R homozygous individuals. Heterozygous individuals (FcgRIIa-HR) have effector cells bearing both allotypes of the receptor and are referred to as “intermediate immune responders” regarding FcgR functions. FcgRIIa-R allotype binds also other IgG subtypes weaker than H allotype [23].

The polymorphism in FcgRIIIa, FcgRIIIa-V158F (rs396991), leads also to different binding affinity to IgG between the two different isoforms of the receptor. *FCGRIIIA* gene has a thymine (T) to guanine (G) missense mutation in amino acid position 158 which changes the valine (V) to phenylalanine (F) in the ligand-binding domain of the receptor. The FcgR-V158 allotype has a stronger binding affinity to IgG1, IgG3 and IgG4 than the F form [23]. It is noteworthy that binding of IgG4 is limited only to the V158 allotype of the receptor [22]. FcgRIIIa are found mostly on natural killer cells (NKs), but also on tissue-specific macrophages and on a subset of monocytes, γδ T-cells and DCs. NK-cells are major components of the cellular defense system against foreign or infected tissue and could be especially relevant in the context of granulocyte-macrophage colony-stimulating factor (GM-CSF) coding oncolytic viruses, since increased activity of NK cells through DC recruitment is one of the expected mechanisms of action of the transgene [28]. Tumor cells are known to have often lost their MHC class I protein expression, which is needed to activate cytotoxic T lymphocyte (CTL) –mediated cell killing. NK-cells do not need MHC I antigen presentation to be able to recognize and destroy an infected tumor cell since they express FcgRIIIa receptors that can bind antibodies bound to antigens presented on target cells. Triggering of NK-cell FcgRIIIa induces ADCC and lymphokine production underlining the central role of this receptor in host defense against viral infections and malignancies [23,29]. Also FcgRIIIa on macrophages (e.g. Kuppfer cells in the liver) are thought to play a role in the clearance of circulating immune complexes [22,23].

The role of FcgR polymorphisms in determining the efficacy of immunotherapies is increasingly recognized. Here, we assessed the association of FcgRIIa and FcgRIIIa with clinical response to, and survival post oncolytic adenovirus therapy.

## Materials and methods

### Patient samples

Analysis carried out in the present study are based on a series of 235 individuals (98 males and 137 females; age median 58 years) with advanced solid tumors refractory to available treatment modalities. These patients were treated with oncolytic adenoviruses in an Advanced Therapy Access Program (ATAP). ATAP was regulated by Finnish Medicines Agency (FIMEA) as determined by EU EC/1394/2007. The inclusion and exclusion criteria have been reported elsewhere [30,31]. The analyses reported here have been approved by HUCH (Helsinki University Central Hospital) Operative Ethics committee.

71,9% of patients included in this present study have been treated with GM-CSF-armed viruses, 15,7% with CD40L-armed viruses and 7,2% with both GM-CSF-and CD40L-armed viruses. Other relevant clinical data are shown in Table 1.

**Table 1:** Patient characteristics

***No. of patients***	**235**
*Age (years when treatments started)*	
Range	3-82
Medium	58
*Sex (No. (%))*	
Female	137 (58,3%)
Male	98 (41,7%)
*Cancer type (No. (%))*	
Colorectal, intestinal and anal	42 (17,9%)
Ovarian (also tubal)	35 (14,9%)
Breast	29 (12,3%)
Sarcomas	21 (8,9%)
Pancreatic and papilla vater	20 (8,5%)
Lung	18 (7,7%)
Neuroblastoma	14 (6,0%)
Prostate	12 (5,1%)
Skin and melanomas	11 (4,7%)
Liver and mesothelioma	9 (3,8%)
Gastric	6 (2,6%)
Biliary tract or cholangio	5 (2,1%)
Urinary tract or bladder	4 (1,7%)
Cervix and endometrial	3 (1,3%)
Renal	3 (1,3%)
Thyroid, thymus or parathydoid	2 (0,9%)
Esophageal	1 (0,4%)

Peripheral blood samples were originally collected from patients to assess presence of virus (biosafety, efficacy and safety implications). Since receptor polymorphisms could also impact safety or efficacy, the same samples were used for FcgR SNP genotyping with Ethics committee permission.

### DNA extraction

Genomic DNA was extracted from patient clot samples by using a QiAmp Blood Mini Kit (Qiagen, Germany). First the samples were thawed in a 37°C water bath and then the clots were transferred into clot spin basket filters (Qiagen, Germany). Samples were spun through the filter by centrifugation (2000 rcf, 5 min., RT). Qiagen QiAmp Blood Mini Kit DNA extraction protocol was used for the DNA extraction. DNA was eluted in nuclease free water (Amresco LLC, Solon, OH, USA) and stored in-20°C freezer until genotype analysis. DNA concentrations were measured by using NanoDrop ND-1000 (Thermo Fisher Scientific, Wilmington, DE, USA). For genotype analyses DNA samples were diluted in nuclease free water (Amresco) to get a final concentration of 1 ng/μl.

### Genotyping

Patients were genotyped for two different Fc gamma receptor polymorphisms, FcgRIIa-H131R (rs1801274) and FcgRIIIa-V158F (rs396991) using TaqMan technology [32] on Applied Biosystems (AB) 7500 Fast Real-Time PCR system (Applied Biosystems Inc., CA, USA). Probes and primers (TaqMan SNP Assays for rs1801274 and rs396991) were ordered from Applied Biosystems. Genotyping was performed by manufacturer’s instructions. Briefly, polymerase chain reactions were prepared in MicroAmp Fast Optical 96-well Reaction Plates (AB) in final volume of 25 μl. The reaction mixes consisted of 2× TaqMan Genotyping Master Mix (AB) and 20× TaqMan SNP Assay Mix (AB) for either FcgRIIa or FcgRIIIa polymorphism. 10 ng of genomic template DNA sample was added per well. Each sample was set up as triplicate. Nuclease free water (Amresco) was used as No Template Control (NTC). Following PCR program was used: initiation at 60°C for 2 minutes and AmpliTaq Gold Enzyme activation at 95°C for 10 minutes followed by 45 cycles of denaturation at 92°C for 15 seconds and annealing and extension at 60°C for 1 minute, allelic discrimination plate read was performed at 60°C for 1 minute. The FcgR genotypes were determined using Allelic Discrimination protocol in the Sequence Detection System (SDS) software provided by Applied Biosystems.

### Statistical analysis

The Pearson’s Chi-square (χ^2^) test was used to asses Hardy-Weinberg equilibrium of genotype and allele frequencies by using OEGE (Online Encyclopedia for Genetic Epidemiology studies) Hardy-Weinberg equilibrium calculator [33]. CubeX (Cubic Exact Solution) software [34] and EM (expectation-maximisation) algorithms [35] were used for analyzing linkage disequilibrium between the two polymorphic loci. The χ^2^ test was used to compare outcomes of the patients according to FcgR polymorphisms. Outcome data were divided to disease control (DC) (i.e. stable disease or better) or to progressive disease (PD) according to patients’ imaging (RECIST or PET criteria [36]) and tumor marker data. Kaplan-Meier survival estimations [37] and log-rank statistics [38] were used to determine the differences in the overall survival (OS) and median time to death. OS was calculated from the date of initiation of the adenovirus therapy to the date of death or to the date of last follow-up when data were censored. The statistical data were obtained by using IBM SPSS Statistics 20 software for Windows (SPSS Inc., Chicago, IL, USA). All *P* values are two-sided and considered statistically significant when ≤ 0,05.

## Results

### Genotypic frequencies of polymorphisms

DNA from 235 patients with advanced cancers refractory to conventional therapies treated with oncolytic virotherapy were genotyped for FcgRIIa-H131R and FcgRIIIa-V158F polymorphisms by TaqMan-based qPCR. Genotyping was successful for all patients. Frequencies of FcgRIIa and FcgRIIIa polymorphisms did not differ significantly from the expected ratios of Hardy-Weinberg equilibrium with χ^2^ = 0,72 (*P* < 0,5) and χ^2^ = 0,01 (*P* < 0,9), respectively. By using a 2-locus linkage disequilibrium analysis for these two polymorphic receptor loci, some linkage disequilibrium (LD), i.e. non-random distribution of alleles, was observed (D’ = 0,28; *P* < 0,01). The observed frequencies and results of LD analysis are shown in Additional file 1: Table S1.

### Analyses of patient survival and treatment responses according to FcgRIIa and FcgRIIIa genotypes

**Figure 1:**
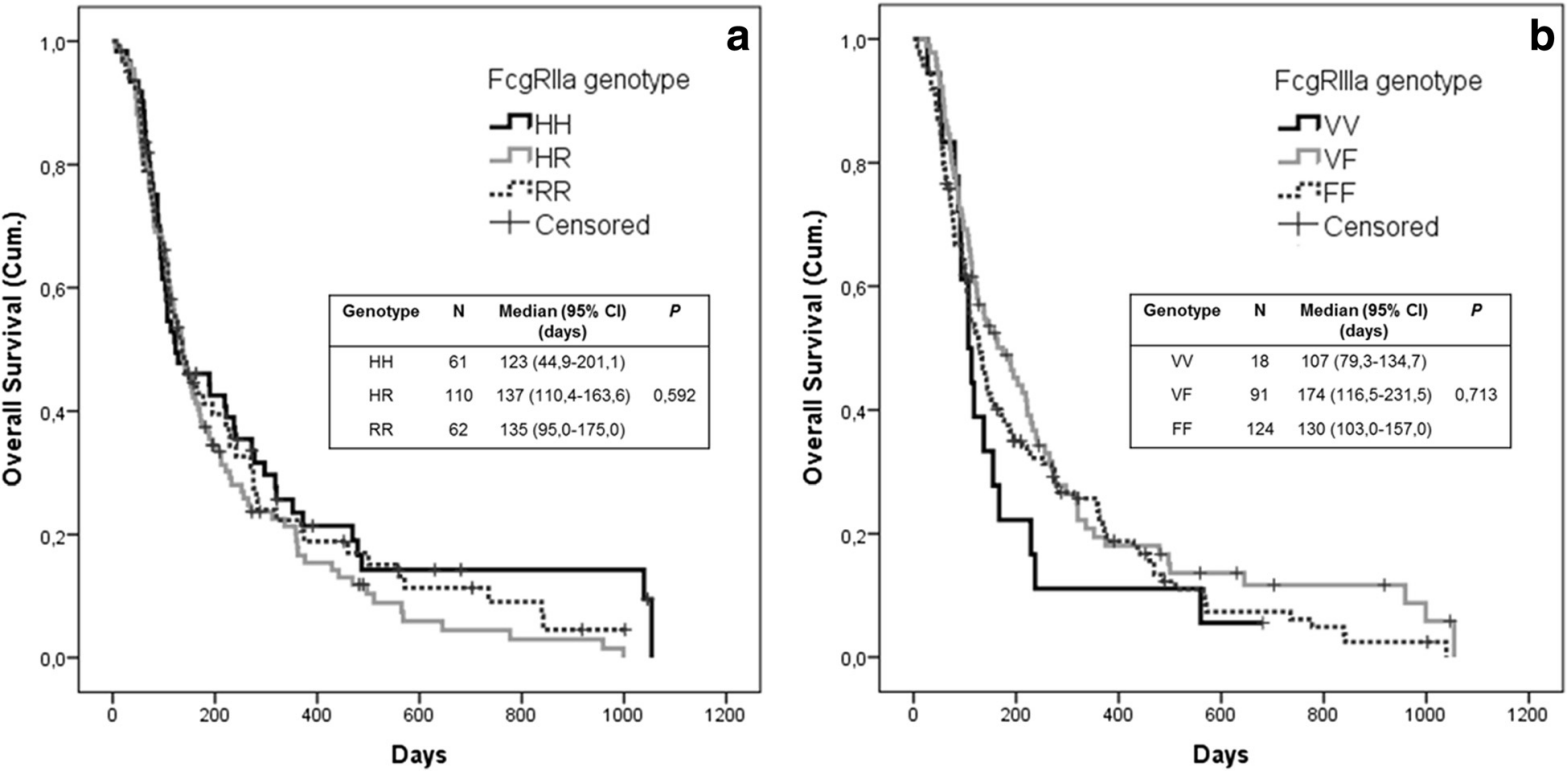
**FcgRIIa or FcgRIIIa genotypes alone are not prognostic of patient survival.** Kaplan-Meier estimates of overall survival by **(a)** FcgRIIa-H131R and **(b)** FcgRIIIa-V158F genotypes. Survival time is presented in days after the first treatment with oncolytic adenovirus. Censored refers to patients who were still alive at the time of performing the analysis.

The median overall survival (OS) time after the first treatment with oncolytic adenoviruses of the studied patients was 130 days. We performed survival estimations for the different genotypes of both FcgRIIa-H131R and FcgRIIIa-V158F polymorphisms by using the Kaplan-Meier analysis to assess whether some of the genotypes would be prognostic of prolonged survival of patients treated with oncolytic adenoviruses. We did not observe any statistically significant differences in OS based on FcgRIIa/IIIa genotypes (FcgRIIa HH vs. HR vs. RR, *P* = 0.335; FcgRIIIa VV vs. VF vs. FF, *P* = 0.193) (Figure 1). In addition, no significant difference in the distribution of clinical responses (disease control (DC) or progressive disease (PD)) was observed between patients with either FcgRIIa HH, HR or RR genotype (42,1%/57,9% *vs.* 43,7%/56,3% *vs.* 37,5%/62,5%; *P* = 0,842). Similarly, no statistically significant difference in the clinical responses was observed between patients with either FcgRIIIa VV, VF or FF genotype (45,5%/54,5% *vs.* 37,9%/62,1% *vs.* 44,6%/55,4%; *P* = 0,730) (Additional file 1: Figure S1). Thus, there was no difference in survival or disease control in association with any of the genotypes individually.

### Analyses of patient survival according to combinations of FcgRIIa and FcgRIIIa genotypes

**Figure 2:**
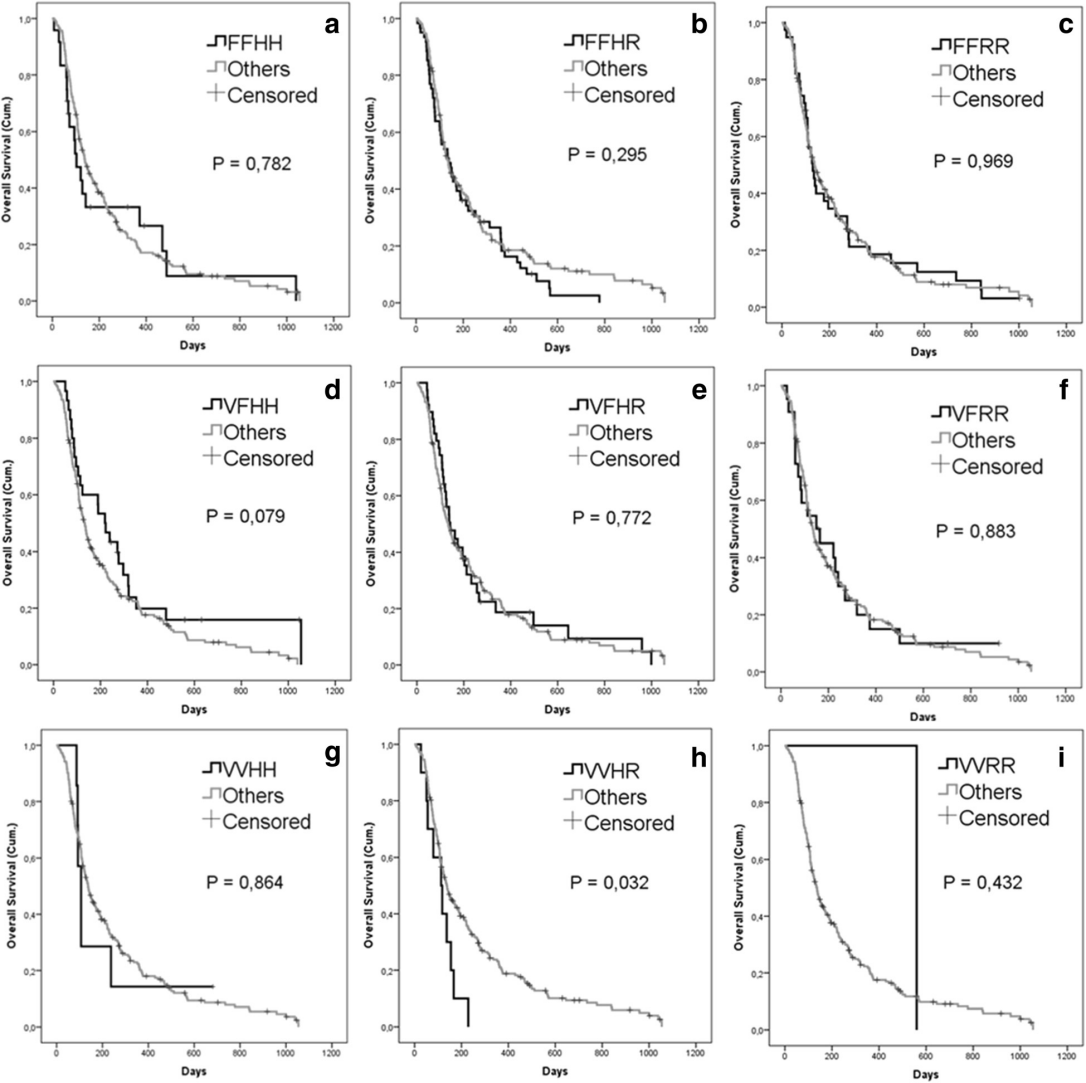
**Survival estimates of all FcgR genotype combinations.** Kaplan-Meier estimate of overall survival of patients plotted by each FcgR genotype combination versus others. **(a)** FFHH (n = 24), **(b)** FFHR (n = 61), **(c)** FFRR (n = 39), **(d)** VFHH (n = 30), **(e)** VFHR (n = 39), **(f)** VFRR (n = 22), **(g)** VVHH (n = 7), **(h)** VVHR (n = 10), **(i)** VVRR (n = 1). The VVHR combination stands out with the poorest survival estimate and VFHH combination with the best estimate, however VVHR vs. others is the only comparison giving a statistically significant difference (*P* = 0,032). Total N of patients included in the analyses is 233.

Because it is known that FcgRs have different expression patterns and biologic significance in different cell types, all of which could contribute to the overall immunological response, we analyzed the combinations of FcgR genotypes (see Additional file 1: Table S2 for observed genotype combinations). We assessed the survival estimations for the different genotype combinations of FcgRIIa-H131R and FcgRIIIa-V158F polymorphisms by using the Kaplan-Meier analysis (Figure 2). Interestingly, one genotype combination, FcgRIIIa-VV and FcgRIIa-HR (VVHR), stood out as a prognostic factor for poor survival after oncolytic adenovirus treatments (*P* = 0,032). Additionally, FcgRIIIa-VF and FcgRIIa-HH (VFHH) genotype combination displayed a borderline trend towards good overall survival, although the correlation was not significant (*P* = 0,079).

### The VVHR genotype combination is prognostic and predictive of long survival in patients treated with oncolytic adenoviruses

**Figure 3:**
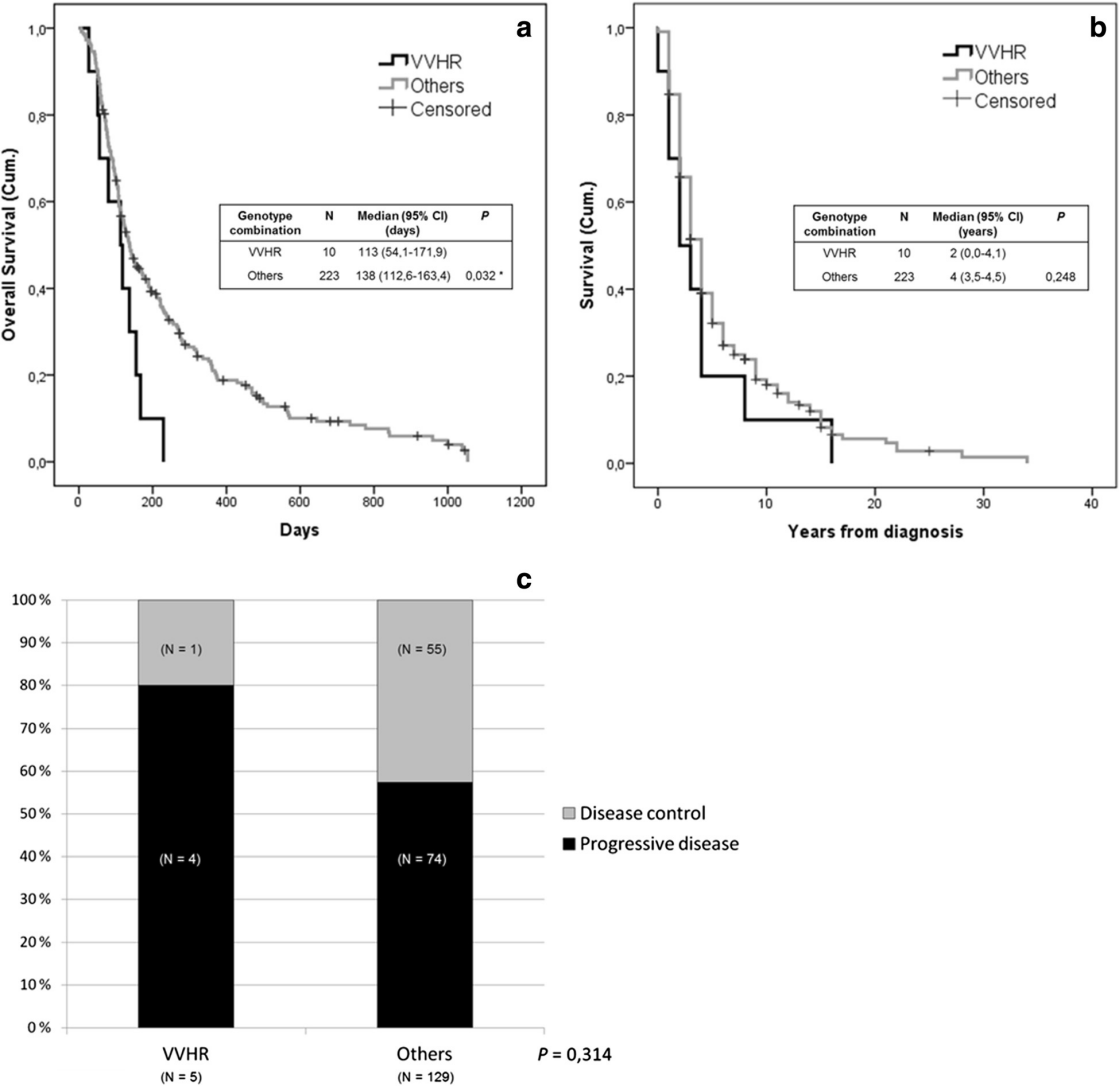
**The VVHR combination of FcgRIIIa and FcgRIIa genotypes shows prognostic and predictive utility in oncolytic adenovirus therapy. (a)** Kaplan-Meier estimate of overall survival after the first treatment with oncolytic adenovirus until death or the end of follow up was plotted by VVHR genotype combination versus all others. The overall survival of patients with VVHR genotype combination is significantly (*P* = 0,032) lower than the one of patients with any other genotype combination. **(b)** Kaplan-Meier estimate of patient survival from the year of cancer diagnosis until the year of death or last follow up was plotted by VVHR genotype combination versus all other combinations reveals that the genotype is not prognostic in cancer patients per se, but only in the context of oncolytic virotherapy. **(c)** Clinical outcome of patients with VVHR genotype combination versus all others. Disease control means stable disease or better in imaging of patients who were progressing prior to therapy. Objective clinical outcome according to RECIST or PET criteria could be determined for 134 patients. VVHR is a combined genotype of homozygotism for V allele of FcgRIIIa and heterozygotism for FcgRIIa. Others represent all the remaining patients without FcRIIIa-158VV plus FcRIIa-131HR genotype combination. Censored refers to patients who were still alive at the time of performing the analysis. Abbreviations: H, histidine allele of FcgRIIa; R, arginine allele of FcgRIIa; V, valine allele of FcgRIIIa; F, phenylalanine allele of FcgRIIIa; CI, confidence interval.

In the Kaplan-Meier survival analyses we observed that patients with a VVHR genotype combination had an overall survival (time from the first virus treatment) estimate significantly shorter than patients with any other genotype combinations with a median of 113 (95% CI: 54,1-171,9) versus 138 (95% CI: 112,6-163,4) days, respectively (Figure 3a). When the survival after cancer diagnosis was compared between patients with VVHR genotype combination and all others, no significant difference could be observed (*P* = 0,248), supporting the notion that the genotype is not prognostic for cancer patients *per se*, but only in the context of oncolytic virotherapy (Figure 3b). Thus, in fact this genotype is not a pure prognostic factor but also predictive of poor survival in patients treated with oncolytic adenovirus. Although the trend was clear, and in accord with the survival result, no statistically significant difference in disease control versus progressive disease was observed between patients with either VVHR or other genotype combinations (20,0%/80,0% *vs.* 42,6%/57,4%; *P* = 0,314) probably due to the limited amount of patients bearing VVHR genotype with an evaluable objective outcome (Figure 3c).

### Different arming molecules have different impacts on survival of patients with different FcgR genotypes

**Figure 4:**
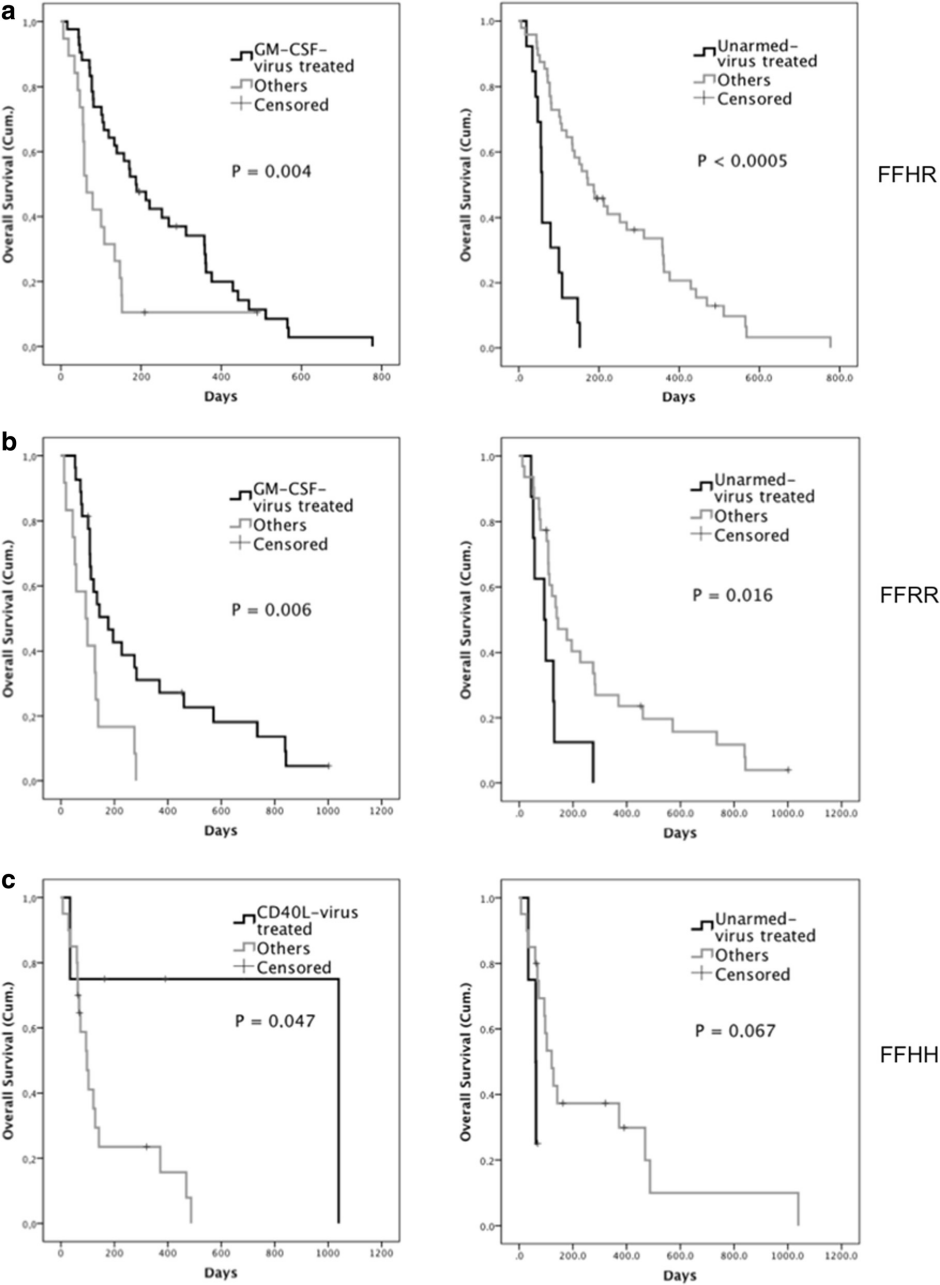
**The effect of virus arming on the survival of patients with FFHR, FFRR and FFHH genotype combinations.** Kaplan-Meier analyses were performed to study the effect of the virus arming (GMCSF, CD40L or unarmed) on survival of patient with FFHR, FFRR and FFHH genotype combinations. Calculations were made by first restricting the study population by each genotype combination and then comparing the overall survival of patient treated with a certain type of virus versus all others. **a)** Survival of patients with FFHR genotype when threated with GM-CSF-armed virus vs. others or with unarmed virus vs. others. **b)** Survival of patients with FFRR genotype when threated with GM-CSF-armed virus vs. others or with unarmed virus vs. others. **c)** Survival of patients with FFHH genotype when threated with CD40L-armed virus vs. others or with unarmed virus vs. others.

GM-CSF and CD40L are both potent recruiters and activators of immune cells and they are also essential for FcgR-mediated effector cell functions. Thus, we studied if the arming molecule present in the virus has also an impact on the survival of patients with different FcgR genotypes (Additional file 1: Table S3) and genotype combinations (Table 2). Overall, patients treated with armed viruses lived longer regardless of genotype. Interestingly, patients with FFHR and FFRR genotypes had significantly longer overall survival after first virus treatment if they were treated with GM-CSF-armed adenovirus, in comparison to other viruses (*P* = 0,004 and *P* = 0,006 respectively) (Figures 4a and 4b). In contrast, treatment of these patients with unarmed virus correlated with shorter survival (*P* < 0,0005 and *P* = 0,016, respectively). Treating FFHH individuals with CD40L-armed virus resulted in longer survival than treatment with other viruses (*P* = 0,047) (Figure 4c).

**Table 2:** The effect of virus arming on the survival of patients with different FcgR genotype combinations

**Genotype**		**Treated with GM-CSF virus**			**Treated with CD40L virus**			**Treated with GM-CSF and CD40L viruses**			**Treated with unarmed virus**		
	**N (all patients)**	**N**	***P***	**+ or -**	**N**	***P***	**+ or -**	**N**	***P***	**+ or -**	**N**	***P***	**+ or -**
FFHH	24	17	0,783	+/−	4	0,047	+	2	0,034	+	4	0,067	-
FFHR	61	42	0,004	+	9	0,106	+	4	0,222	+	13	<0,0005	-
FFRR	39	27	0,006	+	6	0,175	+/−	2	0,166	+	8	0,016	-
VFHH	30	22	0,194	+	6	0,182	+	5	0,103	+	7	0,283	-
VFHR	39	28	0,093	+	10	0,331	+	4	0,025	+	5	0,179	-
VFRR	22	17	0,061	+	2	NA		0	NA		3	0,122	-
VVHH	7	7	NA		0	NA		0	NA		0	NA	
VVHR	10	8	0,081	+	0	NA		0	NA		2	0,081	-
VVRR	1	1	NA		0	NA		0	NA		0	NA	

P = log rank, + or = benefits or disadvantageous (= better or worse OS estimate) when compared to others.

Kaplan-Meier analyses were performed to study the effect of the virus arming (GM-CSF, CD40L, both or unarmed) on survival of patient with different genotype combinations. Calculations were made by first restricting the study population by each genotype combination and then comparing the overall survival of patient treated with a certain type of virus versus all others. Abbreviations: *H* histidine allele of FcgRIIa, *V* valine allele of FcgRIIIa, *F* phenylalanine allele of FcgRIIIa, CD40L, CD40 ligand, *GM-CSF* granulocyte macrophage colony-stimulating factor.

## Discussion

Oncolytic virotherapy has been proposed a potent approach for treatment of advanced cancers. However, it is becoming increasingly recognized that there is variance in efficacy between treated patients; Some patients seem to respond better to the therapy than others. A similar phenomenon has also been observed with some other immunotherapeutics, including monoclonal antibodies [17] and cancer vaccines [12,13]. As immunological aspects have been reported important for the efficacy of oncolytic viruses [5,21,39,40], we hypothesized that there may be some germline differences in the immunological mechanisms of patients that might in part explain differences in response to oncolytic virotherapy. One of these could be Fc gamma receptors (FcgRs), which are known key players in the immune defense against infections and malignancies [23]. Single nucleotide polymorphisms in these receptors lead to different binding affinity of IgG between the receptor allotypes and therefore to varying efficiency in immune defense mechanisms between individuals. FcgR polymorphisms have recently been associated with disease severity of multiple immunological diseases [22,23] and responsiveness of immunological cancer therapies [10,11], although there are also reports suggesting lack of association [41]. To date there are also many conflicting results about which genotype predicts the best outcome or survival [16,18,19], however, this might in fact only reflect the fact that different therapies work in different ways so that there will not be a “universal” genotype (marker) that would predict response to all therapies. In this study, we wanted to asses if FcgR polymorphisms have an effect on survival and/or outcome of cancer patients treated with oncolytic adenoviruses and if these polymorphisms could serve as prognostic and/or predictive biomarkers for oncolytic virotherapy to further enable better selection of patients suitable for this novel therapy.

Our analyses included 235 cancer patients that have been treated with oncolytic adenoviruses as an experimental therapy after all other treatment options had failed. We genotyped all patients for two SNPs in FcgR genes, FcgRIIa-H131R and FcgRIIIa-V158F, using TaqMan-based technology. The distribution of FcgRIIa and FcgRIIIa genotypes observed in our study population were relatively similar to those reported previously for Caucasian [18,42-46] and Finnish [47] populations. This confirms the reliability of the genotyping performed in this study. We observed some linkage disequilibrium between the genotypes, which has also been reported in some previous studies in Dutch [42] and Spanish [48] populations.

It is obvious that FcgRs function in concert with each other and with other receptors, especially in the context of a therapy such as oncolytic viruses which are active on several immunological levels. The overall effect of different FcgRs on clinical outcome is currently poorly understood, but simplistic explanations include proposals that different classes of FcgRs mediate binding by different cell types. For example, it has been proposed that FcgRII is important in the context of antigen presenting macrophages, while FcgRIII is relevant for activity of NK cells [22]. Thus, we were encouraged to investigate the genotype combinations and how they correlate with patient survival and treatment outcome.

In survival estimations one genotype combination (FcgRIIIa-VV + FcgRIIa-HR) stood out as a prognostic factor for poor overall survival after oncolytic virus therapy. Since this genotype did not correlate with length of survival from cancer diagnosis, we hypothesize that this is not a purely prognostic factor but in fact predictive of long survival in patients treated with oncolytic adenoviruses. The small proportion of patients with this genotype combination may limit its clinical utility and therefore our findings may be more interesting from a mechanism-of-action point of view. We also observed another FcgR genotype combination, VFHH (heterozygote for FcgRIIIa and homozygote for FcgRIIa-H allotype), to have a trend towards good overall survival, but the correlation was not significant. Interestingly though, this genotype combination was an “opposite” genotype for the poor responder genotype VVHR (homozygote for FcgRIIIa-V allotype and heterozygote for FcgRIIa) supporting a possible mechanistic aspect.

We observed that different arming molecules associated differently with survival in patients with different FcgR genotype combinations. This is not very surprising since GM-CSF and CD40L are both potent recruiters and activators of immune cells (e.g. DCs and NK cells) essential for FcgR-mediated effector cell functions. Thus, individuals with FFHR and FFRR genotypes might have lower innate activity of NK cells, and therefore their tumors feature less resistance to NK cell mediated killing. When a potent NK recruitment signal is provided by virally produced GM-CSF, anti-tumor efficacy may ensue. Importantly, since the effect of GM-CSF is paracrine, activity of the recruited NK-cells is not only against infected cells, but also against “bystanders”. If the baseline NK activity is low, perhaps infected cells are not cleared as rapidly as when baseline NK activity is high, as predicted for VVHR. These hypotheses are in accord with VVHR correlating with poor survival, as this genotype might indicate high NK responsiveness against infected cells, limiting virus dissemination (Additional file 1: Figure S2).

It has also been shown that many inflammatory molecules, including cytokines and growth factors, can alter FcgR expression and FcgR-mediated immune responses, although not all reports agree. Pleiotropic effects of these inflammatory molecules have been shown to act locally to distract and downregulate FcgR-mediated immune functions [21,26,47,48]. On the other hand, e.g. GM-CSF and IFN-γ have been shown to increase the activity and expression of FcgRIIa and FcgRIIIa (49–52). Possibly these features of GM-CSF and CD40L contributed to long survival in individuals with FFHR and FFRR genotypes (GM-CSF) or FFHH (CD40L); lower baseline activity was compensated by transgene products resulting in immunological activation.

NK cells have been proposed limiting for the efficacy of oncolytic viruses and they are the main cell type expressing FcgRIIIa [49]. NK cells can mediate antibody-dependent cell killing by ADCC, which occurs when the FcgRIIIa molecules on effector cells are cross-linked by binding to the IgG molecules that are present on the target cell [23]. In the case of homozygous individuals for FcgRIIIa-V allotype (VV), infected tumor cells are probably killed efficiently by these “strong binding” receptor variants, possibly before the virus has even had time to replicate effectively. This may result in fierce anti-viral ADCVI (antibody-dependent cell-mediated virus inhibition) [9] against oncolytic adenoviruses partially inhibiting its efficacy.

In line with this thinking, it is logical that intermediate binding of NK cells would be optimal, as ADCVI would not be too prominent, but that infected tumor cells – and bystander cells - would be eventually eradicated. In other words, the virus has time to replicate and spread while simultaneously the NK cells are still effective enough to kill the tumor cells. This would result in individuals heterozygous for FcgRIIIa polymorphism (VF) benefiting the most. There is always balancing between ADCC and ADCVI that are partly overlapping and competing events in the context of oncolytic virus therapy [9].

FcgRIIa receptors are widely distributed on many immune cell types, but are mostly considered to be prominent on phagocytic cells, including tissue macrophages, the most important class of antigen presenting cells (APC) [50]. Given the mechanism-of-action of oncolytic adenoviruses armed with immunostimulatory molecules such as GM-CSF, effective recognition of tumor epitopes by APCs seems likely to be important for efficacy and subsequent survival. Therefore, it is logical that we observed the trend for increased survival in HH-homozygotes, which can strongly bind IgG and a significant correlation with survival in CD40L virus treated patients.

## Conclusions

According to the results of this study, we suggest that FcgR-mediated blockage of virus infectivity via degradation of immune complexes in APCs and ADCC together with ADCVI could have an effect on oncolytic virotherapy and therefore on the therapy responsiveness. At this point, our understanding of the distribution of the different classes of FcgR on different immunological cells is insufficient to allow drawing of firm conclusions. Moreover, the interplay of different cell types in the context of response to oncolytic virotherapy are poorly understood. Also, due to relatively small and heterogeneous study cohort no definitive conclusions can be drawn from these results and should thus be taken as hypothesis generating rather than conclusive analysis. However, our empiric observation that polymorphisms in FcgRIIa and FcgRIIIa seem to have potential as prognostic and predictive biomarkers for oncolytic adenovirus therapies could eventually enable selection of patients responsive to the treatments. Importantly, this observation was made in real-life human patients treated with oncolytic adenoviruses. The results presented here are, according to our knowledge, the first associations studied between Fc gamma receptors and survival of oncolytic virus treated patients. Our data set the stage for prospective study of these genotypes in the context of trials with oncolytic adenoviruses coding for immunostimulatory molecules. Since many different oncolytic viruses are currently being used, the phenomenon of oncolysis might nevertheless have common features, and the most popular transgenes employed tend to be similar, often featuring immunostimulatory mechanisms of actions. Thus, our data might have relevance for the entire field of cancer therapy with oncolytic viruses. Moreover, as immunological aspects are daunting to study in the laboratory, especially in the context of species-specific agents such as oncolytic adenoviruses, human data could provide important mechanistic data.

### Consent

Written informed consent was obtained from the patient for the publication of this report.

## Abbreviations

ADCC: Antibody-dependent cellular cytotoxicity; ADCVI: Antibody-dependent cell-mediated virus inhibition; APC: Antigen presenting cell; CD40L: CD40 ligand; DC: Dendritic cell; FcgR: Fc gamma receptor; GM-CSF: Granulocyte macrophage colony-stimulating factor; ITAM: Immunoreceptor tyrosine-based activation motif; ITIM: Immunoreceptor tyrosine-based inhibition motif; MHC: Major histocompatibility complex; NK: Natural killer cell.

## Competing interests

A.H. is shareholder in and consultant to Oncos Therapeutics, Ltd.

## Authors’ contributions

MH carried out the design of the study, acquisition, analysis and interpretation of the data, statistical analyses and drafted the manuscript. RH and MO helped with patient data analyses. SP helped with DNA extractions and genotyping. IL wrote the application for Operative Ethics committee and provided advice with patient data. TJ provided the patient material. AK provided scientific advice and help with patient data. VC and AH contributed to conception and design of the study, provided scientific advice and help with manuscript writing. All authors read and approved the final manuscript.

## Supplementary Material

Additional file 1: Table S1 Observed frequencies of polymorphisms and linkage disequilibrium statistics. **Table S2**. Observed frequencies of genotype combinations. **Table S3**. The effect of virus arming on the survival of patients with different FcgR genotypes. Kaplan-Meier analyses were performed to study the effect of the virus arming (GM-CSF, CD40L, both or unarmed) on survival of patients with different genotypes. Calculations were made by first restricting the study population by each genotype and then comparing the overall survival for patients treated with a certain type of virus versus all other patients. Abbreviations: H, histidine allele of FcgRIIa; V, valine allele of FcgRIIIa; F, phenylalanine allele of FcgRIIIa; CD40L, CD40 ligand; GM-CSF, granulocyte macrophage colony-stimulating factor. **Figure S1**. FcgRIIa and FcgRIIIa genotypes are not predictive of imaging results in patients treated with oncolytic adenovirus therapy. Clinical outcome of patients treated with oncolytic adenoviruses by (a) FcgRIIa-H131R and (b) FcgRIIIa-V158F genotypes. Objective clinical outcome could be determined for 134 patients. Abbreviations: DC, disease control (= stable disease or better); PD, progressive. **Figure S2**. Hypothetical mechanisms-of-action. (a) Strong binding of NK cells to tumor cell-bound IgG (VV) causes virus elimination prior to effective oncolytic dissemination. (b) Intermediate activity of NK cells (VF) gives time for the virus to replicate and spread while simultaneously being still effective enough in tumor cell killing. This combined with efficient tumor antigen presentation by APCs (HH) plus the ability of GM-CSF and CD40L to recruit more APCs and other immune cells to the tumor site may explain the good responsiveness to oncolytic adenovirus therapy with armed viruses. Abbreviations: APC, antigen presenting cell; NK cell, natural killer cell; MHC-II, major histocompatibility complex II. Click here for file
