# Supplementary material for: Fc-gamma receptor polymorphisms as predictive and prognostic factors in patients receiving oncolytic adenovirus treatment
Source: J Transl Med. 2013 Aug 21;11:193. doi: 10.1186/1479-5876-11-193 (PMC3765225; doi:10.1186/1479-5876-11-193)

Supplementary data

Supplementary figures and figure legends:

Table S1. Observed frequencies of polymorphisms and linkage disequilibrium statistics.

Allele frequencies				Genotype frequencies						Haplotype frequencies				LD statistics			
FcgRIIa		FcgRIIIa		FcgRIIa			FcgRIIIa										
H	R	V	F	HH	HR	RR	VV	VF	FF	HV	HF	RV	RF	D´	r^2	χ^2	*P*
0,500	0,500	0,274	0,726	0,264	0,472	0,264	0,077	0,396	0,528	0,175	0,325	0,099	0,401	0,278	0,0293	6,89	< 0,01

Abbreviations: FcgR, fragment c gamma receptor; H, FcgRIIa histidine allele; R, FcgRIIa arginine allele; V, FcgRIIIa valine allele; F, FcgRIIIa phenylalanine allele; LD, linkage disequilibrium; D´, LD/LDmax (LD is complete when D´=1); r^2, correlation coefficient (LD is perfect when $r^2 = 1$); χ^2 , chi square.

Table S2. Observed frequencies of genotype combinations.

	FcgRIIIa-V158F		
FcgRIIa-H131R	VVHH 7	VFHH 31	FFHH 24
	VVHR 10	VFHR 40	FFHR 61
	VVRR 1	VFRR 22	FFRR 39

Figure S1. FcgRIIa and FcgRIIIa genotypes are not predictive of imaging results in patients treated with oncolytic adenovirus therapy. Clinical outcome of patients treated with oncolytic adenoviruses by **(a)** FcgRIIa-H131R and **(b)** FcgRIIIa-V158F genotypes. Objective clinical outcome could be determined for 134 patients. Abbreviations: DC, disease control (= stable disease or better); PD, progressive disease; H, histidine allele of Fc gamma receptor IIa; R, arginine allele of FcgRIIa; V, valine allele of FcgRIIIa; F, phenylalanine allele of FcgRIIIa.

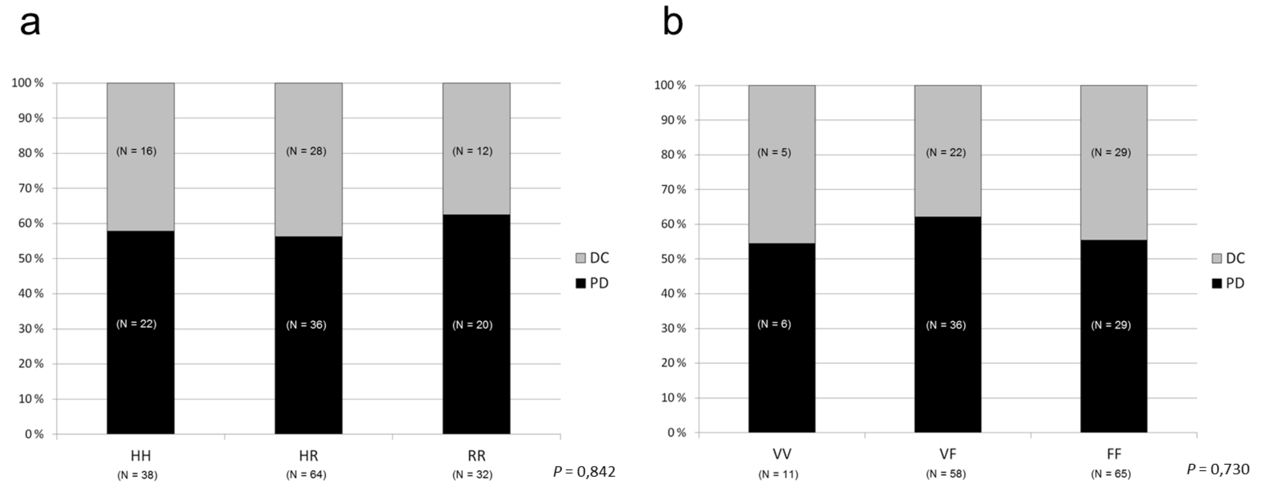

Table S3. The effect of virus arming on the survival of patients with different FcgR genotypes. Kaplan-Meier analyses were performed to study the effect of the virus arming (GM-CSF, CD40L, both or unarmed) on survival of patients with different genotypes. Calculations were made by first restricting the study population by each genotype and then comparing the overall survival for patients treated with a certain type of virus versus all other patients. Abbreviations: H, histidine allele of FcgRIIa; V, valine allele of FcgRIIIa; F, phenylalanine allele of FcgRIIIa; CD40L, CD40 ligand; GM-CSF, granulocyte macrophage colony-stimulating factor.

Genotype		Treated with GM-CSF virus			Treated with CD40L virus			Treated with both GM-CSF and CD40L viruses			Treated with unarmed virus		
	N (all patients)	N	*P*	+ or -	N	*P*	+ or -	N	*P*	+ or -	N	*P*	+ or -
HH	61	46	0,238	+	10	0,025	+	7	0,011	+	11	0,102	-
HR	110	78	0,001	+	19	0,010	+	8	0,004	+	20	<0,0005	-
RR	62	45	0,001	+	8	0,884	+/-	2	0,218	+	11	0,004	-
VV	18	16	0,004	+	0	NA		0	NA		2	0,765	+
VF	91	67	0,004	+	18	0,086	+	9	0,003	+	15	0,173	+
FF	124	86	<0,0005	+	19	0,014	+	8	0,004	+	25	0,664	+/-

P = log rank, +or- = beneficial or disadvantageous (= better or worse OS estimate) when compared to others.

Figure S2. Hypothetical mechanisms-of-action. (a) Strong binding of NK cells to tumor cell-bound IgG (VV) causes virus elimination prior to effective oncolytic dissemination. **(b)** Intermediate activity of NK cells (VF) gives time for the virus to replicate and spread while simultaneously being still effective enough in tumor cell killing. This combined with efficient tumor antigen presentation by APCs (HH) plus the ability of GM-CSF and CD40L to recruit more APCs and other immune cells to the tumor site may explain the good responsiveness to oncolytic adenovirus therapy with armed viruses. Abbreviations: APC, antigen presenting cell; NK cell, natural killer cell; IgG, immunoglobulin G; FcgR, fragment c gamma receptor; H, histidine allele; R, arginine allele; V, valine allele; F, phenylalanine allele; MHC-II, major histocompatibility complex II.

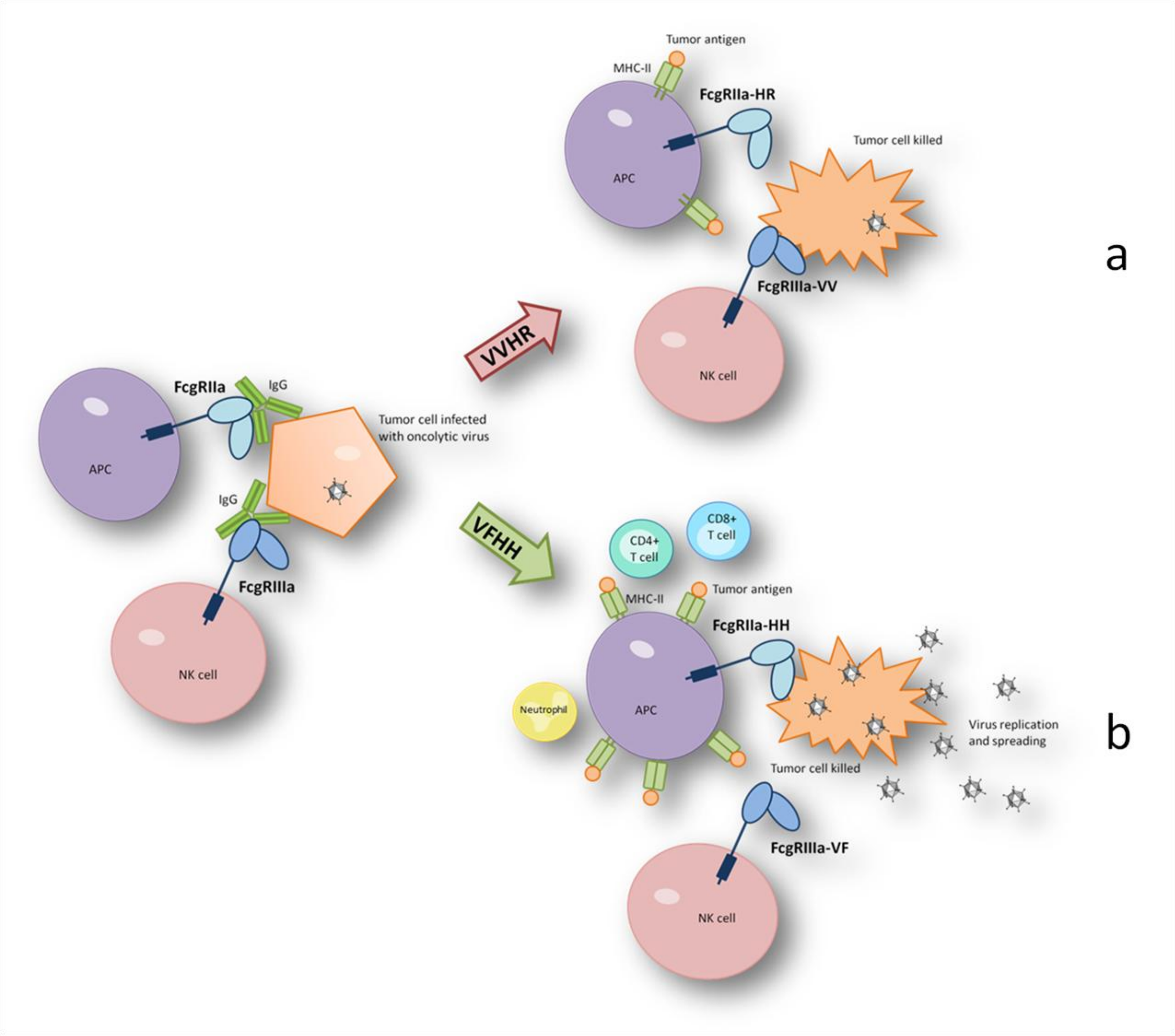

Supplement: Additional file 1: Table S1 — Observed frequencies of polymorphisms and linkage disequilibrium statistics. Table S2. Observed frequencies of genotype combinations. Table S3. The effect of virus arming on the survival of patients with different FcgR genotypes. Kaplan-Meier analyses were performed to study the effect of the virus arming (GM-CSF, CD40L, both or unarmed) on survival of patients with different genotypes. Calculations were made by first restricting the study population by each genotype and then comparing the overall survival for patients treated with a certain type of virus versus all other patients. Abbreviations: H, histidine allele of FcgRIIa; V, valine allele of FcgRIIIa; F, phenylalanine allele of FcgRIIIa; CD40L, CD40 ligand; GM-CSF, granulocyte macrophage colony-stimulating factor. Figure S1. FcgRIIa and FcgRIIIa genotypes are not predictive of imaging results in patients treated with oncolytic adenovirus therapy. Clinical outcome of patients treated with oncolytic adenoviruses by (a) FcgRIIa-H131R and (b) FcgRIIIa-V158F genotypes. Objective clinical outcome could be determined for 134 patients. Abbreviations: DC, disease control (= stable disease or better); PD, progressive. Figure S2. Hypothetical mechanisms-of-action. (a) Strong binding of NK cells to tumor cell-bound IgG (VV) causes virus elimination prior to effective oncolytic dissemination. (b) Intermediate activity of NK cells (VF) gives time for the virus to replicate and spread while simultaneously being still effective enough in tumor cell killing. This combined with efficient tumor antigen presentation by APCs (HH) plus the ability of GM-CSF and CD40L to recruit more APCs and other immune cells to the tumor site may explain the good responsiveness to oncolytic adenovirus therapy with armed viruses. Abbreviations: APC, antigen presenting cell; NK cell, natural killer cell; MHC-II, major histocompatibility complex II. [file 1479-5876-11-193-S1.pdf]
